# Diagnostic Pitfalls in Adult-Onset Spinal Muscular Atrophy Type 4: When Incidental Lumbar Stenosis Confounds the Clinical Picture

**DOI:** 10.7759/cureus.108324

**Published:** 2026-05-05

**Authors:** Shamas Rafique, Iqra Rafiq

**Affiliations:** 1 Family Medicine, Family Medical Health Care PLLC, New York, USA; 2 Internal Medicine, First Affiliated Hospital of Xinjiang Medical University, Urumqi, CHN; 3 Medicine and Surgery, BeeWell International Hospital, Islamabad, PAK; 4 General Medicine, Services Hospital Lahore, Lahore, PAK; 5 Medicine and Surgery, First Affiliated Hospital of Xinjiang Medical University, Urumqi, CHN

**Keywords:** adult-onset spinal muscular atrophy, anterior horn cell disease, diagnostic delay, electrodiagnostic studies, genetic testing, incidental mri findings, lumbar spinal stenosis, proximal muscle weakness, smn1 gene deletion, spinal muscular atrophy type 4

## Abstract

Spinal muscular atrophy type 4 (SMA‑4) represents a rare, late‑onset motor neuronopathy with insidious proximal limb weakness and preserved walking ability. Its slow progression and vague initial symptoms often cause years of diagnostic uncertainty. We describe a 38‑year‑old man who experienced slowly worsening, fatigue‑related proximal leg weakness over three years. Initial suspicion fell on a metabolic myopathy, and later, lumbar spine magnetic resonance imaging (MRI) showed multilevel degenerative changes with canal stenosis, findings that were initially attributed to the cause but did not align with the clinical picture. Crucially, the patient's younger brother had already received a genetic diagnosis of SMA‑4.

Nerve conduction studies were normal. Needle electromyography (EMG) revealed widespread chronic partial denervation with reinnervation, yet the paraspinal muscles remained electrically silent, a pattern that localizes the lesion to the anterior horn cell. Genetic testing subsequently confirmed homozygous deletion of SMN1 exon 7. This report highlights how incidental imaging abnormalities can confound the diagnostic process and underscores the importance of systematic clinical‑electrodiagnostic‑genetic correlation in adult‑onset proximal weakness, particularly in the era of available disease‑modifying therapies.

## Introduction

Spinal muscular atrophy (SMA) is an autosomal recessive disorder caused by insufficient survival motor neuron (SMN) protein, which leads to the selective loss of spinal alpha motor neurons. The disease was first linked to the SMN1 gene by Lefebvre et al. [1]. Over 95% of cases carry a homozygous deletion of SMN1 exon 7 on chromosome 5q13 [2]. The nearly identical SMN2 gene modifies disease severity through the copy number‑dependent production of functional SMN protein [2,3].

Clinical classification divides SMA into four types according to age of onset and peak motor function. SMA type 4 (SMA-4) begins after 18 years of age; patients usually remain ambulatory and experience slow progression [2,4]. SMA‑4 accounts for fewer than 5% of all SMA cases, and diagnostic delays of several years are typical because the presentation is subtle and mimics more common conditions [5].

Degenerative spine disease is ubiquitous in the adult population, and magnetic resonance imaging (MRI) often reveals abnormalities even in asymptomatic individuals [6]. Consequently, when a patient with proximal weakness is found to have spinal stenosis, there is a strong temptation to attribute the symptoms to that structural finding, even when the clinical pattern is inconsistent. While earlier reports have described delayed diagnosis in the setting of normal imaging [7], this case illustrates a different trap: abnormal but irrelevant imaging that diverts attention from the correct diagnosis.

A key laboratory clue in such cases is the dissociation between elevated creatine kinase (CK) and normal aldolase, which argues against a primary myopathy and suggests a neurogenic process [8-10]. Furthermore, the electrodiagnostic signature of anterior horn cell disease, diffuse limb denervation with spared paraspinal muscles, should prompt expedited genetic testing for SMA [11,12]. The SMN2 copy number further predicts the adult‑onset phenotype, and the correlation between SMN2 copy number and disease severity has been well established [3,13]. Finally, disease‑modifying therapies such as nusinersen and risdiplam have been shown to stabilize or improve motor function in adult SMA patients [14,15].

We present a patient whose SMA‑4 diagnosis was postponed because of multilevel lumbar stenosis seen on MRI, and we discuss how electrodiagnostic studies and targeted family history assessment ultimately resolved the diagnostic dilemma.

## Case presentation

Clinical history

A 38‑year‑old previously active man presented with a three‑year history of gradually progressive, painless weakness in both legs, predominantly affecting the thighs. He had trouble rising from a low sofa and needed to push off with his arms (a Gower‑like maneuver). Climbing stairs became difficult, requiring the handrail. He denied any numbness, tingling, burning pain, or bowel/bladder issues. He also reported no swallowing or speech changes and no shortness of breath.

A critical piece of information emerged during the neuromuscular consultation: the patient's younger brother had recently been diagnosed with genetically proven SMA‑4 after his own lengthy diagnostic journey. This family history had not been pursued earlier.

The patient gave written consent to participate in the case report but declined permission to publish photographs of his legs.

Physical examination

Motor examination revealed symmetric weakness of hip flexion and extension (Medical Research Council grades 4/5 and 4‑/5, respectively). Distal leg muscles (ankle and toe movements) were normal (5/5). Deep tendon reflexes were reduced at both knees and ankles. Sensory testing, coordination, and cranial nerves were intact. No fasciculations, spasticity, or upper motor neuron signs were observed. Notably, the pattern of weakness was non‑dermatomal and symmetric, incompatible with lumbar radiculopathy. Inspection showed visible wasting of the quadriceps and calf muscles.

Laboratory workup

Initial blood tests showed a mildly elevated creatine kinase (CK) of 310 U/L (normal <196 U/L) and a normal aldolase (5.2 U/L; reference up to 8.0 U/L). A comprehensive metabolic panel, thyroid function, HbA1c, vitamin B12, inflammatory markers (C‑reactive protein and erythrocyte sedimentation rate), antinuclear antibody, myositis antibody panel, serum protein electrophoresis, and electrolytes were all normal or negative.

Specific Laboratory Values

Lactate was measured at 1.2 mmol/L (reference range: 0.5-2.2 mmol/L). Pyruvate was 0.08 mmol/L (reference range: 0.03-0.10 mmol/L). Serum electrolytes (sodium, potassium, chloride, and bicarbonate) were 140 mmol/L, 4.1 mmol/L, 102 mmol/L, and 24 mmol/L, respectively, all within normal limits. These findings effectively ruled out inflammatory, endocrine, metabolic, and autoimmune causes of proximal weakness. Table 1 summarizes these results.

**Table 1:** Summary of laboratory findings CK: creatine kinase; TSH: thyroid‑stimulating hormone; ANA: antinuclear antibody; U/L: units per liter; µIU/mL: micro-international units per milliliter; pg/mL: picograms per milliliter; mmol/L: millimoles per liter

Test	Result	Reference range	Interpretation
CK	310 U/L	44-196 U/L	Mild, non‑specific elevation
Aldolase	5.2 U/L	≤8.0 U/L	Normal argues against primary myopathy
TSH	2.1 µIU/mL	0.4-4.5 µIU/mL	Normal
Vitamin B12	485 pg/mL	200-900 pg/mL	Normal
ANA, myositis panel	Negative	Negative	No autoimmune myopathy
Lactate	1.2 mmol/L	0.5-2.2 mmol/L	Normal
Pyruvate	0.08 mmol/L	0.03-0.10 mmol/L	Normal
Sodium	140 mmol/L	135-145 mmol/L	Normal
Potassium	4.1 mmol/L	3.5-5.1 mmol/L	Normal
Chloride	102 mmol/L	98-106 mmol/L	Normal
Bicarbonate	24 mmol/L	22-28 mmol/L	Normal

Imaging

**Figure 1:**
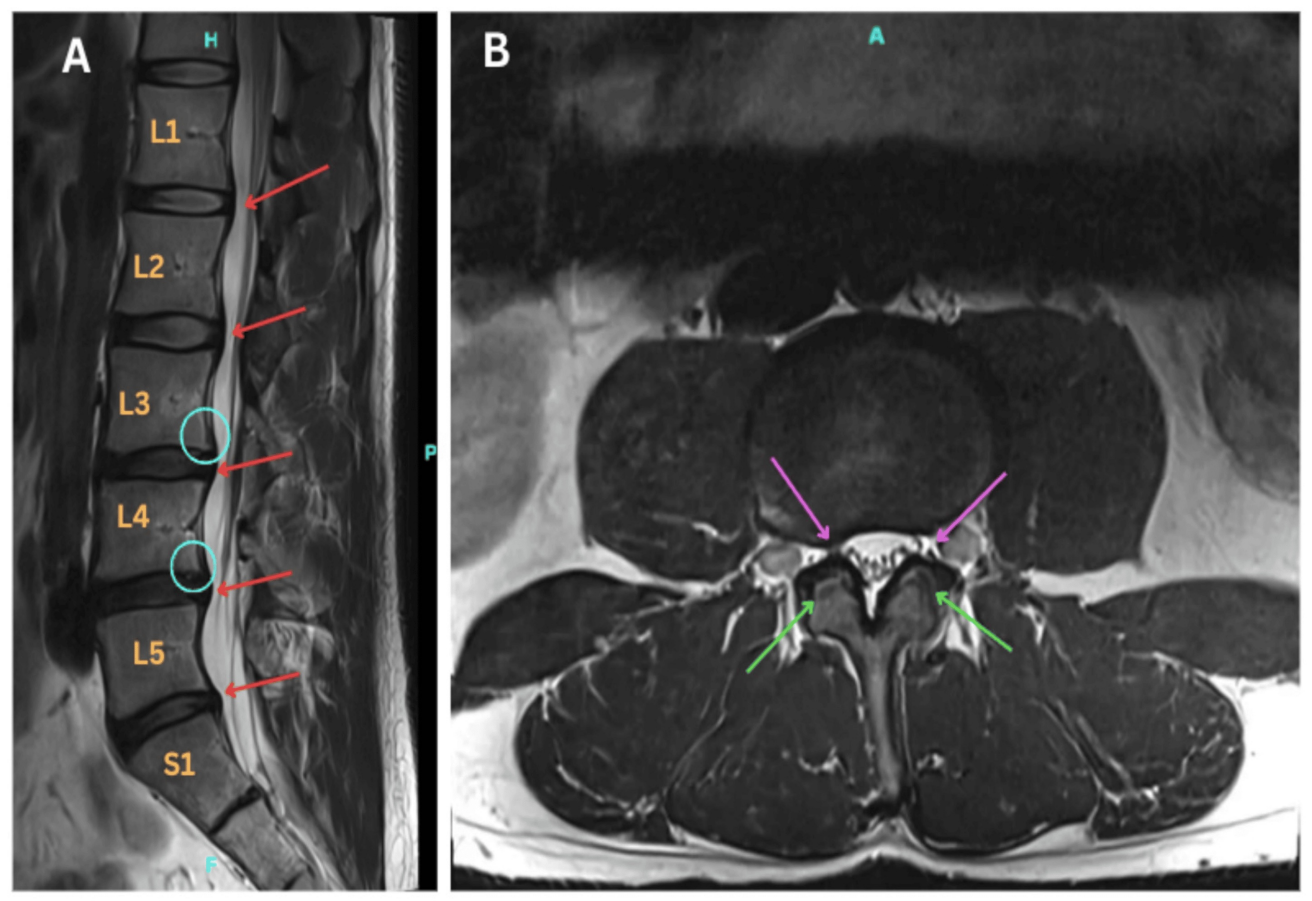
Magnetic resonance imaging of the lumbar spine without contrast (A) Sagittal T2‑weighted image demonstrates multilevel degenerative disc disease; blue circles indicate retrolisthesis at L3‑L4 and L4‑L5, and red arrows mark moderate spinal canal stenosis from L2 through L5. (B) Axial T2‑weighted image at the L4‑L5 level; purple arrows highlight bilateral foraminal narrowing, and green arrows indicate facet arthropathy.

Lumbar spine MRI without contrast (Figure 1) demonstrated mild multilevel degenerative disc disease with disc bulges and facet arthropathy (green arrows, Figure 1B), superimposed on a developmentally narrow spinal canal. There was retrolisthesis of L3 on L4 and L4 on L5 (blue circles, Figure 1A). Central spinal canal stenosis was present at L2‑L3 (mild to moderate), L3‑L4 (moderate), and L4‑L5 (mild to moderate) (red arrows, Figure 1A). Bilateral neural foraminal stenosis was noted at L4‑L5 (moderate to severe on the right, moderate on the left), with additional right‑sided foraminal stenosis at L3‑L4 (moderate) (purple arrows, Figure 1B); these findings implied potential compromise of the right L3 and bilateral L4 nerve roots. The lateral recesses remained patent, and the traversing nerve roots appeared uninvolved. These findings were initially interpreted as the likely cause of the patient's proximal weakness. However, the clinical examination did not match a radiculopathy: weakness was proximal, symmetric, and without sensory loss or radicular pain. This mismatch prompted further neurophysiological evaluation.

Electrodiagnostic studies (electromyography (EMG)/nerve conduction studies (NCS))

NCS of sensory and motor nerves in all four limbs were normal, effectively excluding peripheral neuropathy and neuromuscular junction disorders.

Needle EMG revealed chronic active denervation in proximal lower limb muscles (iliopsoas and vastus lateralis), with fibrillation potentials, positive sharp waves, and large‑amplitude, long‑duration motor unit potentials (MUPs) with reduced recruitment. Distal muscles (tibialis anterior and medial gastrocnemius) showed chronic neurogenic changes without active denervation. Critically, the lumbosacral paraspinal muscles were completely normal, a finding that excludes active radiculopathy and points to a process proximal to the nerve root. Upper limb muscles (deltoid, triceps, and first dorsal interosseous) also demonstrated chronic neurogenic changes, indicating subclinical generalized involvement, while cervical paraspinals remained normal. Table 2 details these findings.

**Table 2:** Summary of needle electromyography findings PSWs: positive sharp waves; MUP: motor unit potential; FDI: first dorsal interosseous

Region	Muscle	Spontaneous activity	MUP morphology	Recruitment	Implication
Proximal leg	Iliopsoas, vastus lateralis	Fibrillations, PSWs	Large, long	Reduced	Active denervation
Distal leg	Tibialis anterior	Rare fibrillations	Large	Mildly reduced	Chronic neurogenic
Distal leg	Medial gastrocnemius	None	Large	Preserved	Chronic neurogenic
Paraspinal	Lumbosacral	None	Normal	Normal	No radiculopathy
Upper limb	Deltoid, triceps, FDI	None	Large	Reduced	Subclinical involvement
Paraspinal	Cervical	None	Normal	Normal	Supports anterior horn cell

The most significant electrodiagnostic finding was the combination of diffuse limb denervation with spared paraspinal muscles, the electrophysiological signature of anterior horn cell disease. This pattern effectively rules out polyradiculopathy and should prompt expedited genetic testing for SMA. Early recognition is particularly important, as diagnostic delay may result in missed opportunities for the timely initiation of disease‑modifying therapy.

Genetic testing

Because the EMG pattern pointed squarely to the anterior horn cell, targeted genetic testing for 5q‑associated SMA was performed. Polymerase chain reaction (PCR)‑based analysis revealed a homozygous deletion of SMN1 exon 7. SMN2 copy number was 3, a genotype strongly linked to the milder, adult‑onset phenotype. These results confirmed the diagnosis of SMA-4, as shown in Table 3.

**Table 3:** Genetic confirmation of SMA SMN1: survival motor neuron 1 gene; SMN2: survival motor neuron 2 gene; SMA-4: spinal muscular atrophy type 4

Gene	Finding	Interpretation
SMN1 exon 7	Homozygous deletion	Confirms 5q‑SMA
SMN2 copy number	Three copies	Consistent with SMA-4

Treatment and follow-up

After genetic confirmation, the patient received genetic counseling and a discussion of available disease‑modifying therapies. He chose to start risdiplam, an oral SMN2 splicing modifier. He also began physical therapy. At the six‑month follow‑up, he reported subjective stabilization of weakness and remained ambulatory without further progression. Long‑term monitoring with serial functional scales and CK measurements is ongoing.

Clinical timeline

Table 4 provides a structured overview of the diagnostic and therapeutic milestones.

**Table 4:** Timeline of diagnostic and therapeutic events CK: creatine kinase; MRI: magnetic resonance imaging; EMG: electromyography; NCS: nerve conduction studies; SMA-4: spinal muscular atrophy type 4

Timepoint	Event
3 years prior to diagnosis	Onset of slowly progressive, painless proximal leg weakness; difficulty rising from low surfaces and climbing stairs
Initial evaluation	Mild CK elevation (310 U/L) with normal aldolase; initial suspicion of metabolic myopathy
Neuromuscular consultation	Younger brother's genetically confirmed SMA‑4 identified; family history previously not pursued
MRI lumbar spine	Multilevel degenerative changes, spinal canal stenosis, and foraminal narrowing: imaging findings initially overemphasized despite clinical mismatch
EMG/NCS	Normal nerve conduction; diffuse chronic limb denervation with completely spared lumbosacral and cervical paraspinal muscles → localizes to the anterior horn cell
Genetic testing	Homozygous deletion of SMN1 exon 7 with three SMN2 copies → confirms SMA-4
Treatment initiation	Risdiplam started concurrent physical therapy
6‑month follow‑up	Subjective stabilization of weakness; remains ambulatory without progression; ongoing monitoring

## Discussion

This case illustrates a common but underrecognized diagnostic trap: the discovery of incidental lumbar spinal stenosis on MRI in a patient with SMA‑4. Because degenerative spine changes are extremely frequent in adults, they can easily be overinterpreted as the cause of proximal weakness, especially when the weakness is bilateral and lower limb predominant. In our patient, the clinical picture of symmetric, non‑dermatomal, painless, purely motor weakness with areflexia was fundamentally incompatible with lumbar radiculopathy, yet the imaging findings were initially overemphasized, delaying the correct diagnosis by years.

A similar phenomenon occurs when MRI is normal: clinicians may be falsely reassured and continue searching for myopathic or metabolic etiologies, as recently reported by Rafique and Odeh [7]. Taken together, these cases demonstrate that MRI findings, whether normal or abnormal, must be interpreted within the clinical context. When the history and examination do not align with a structural lesion, further investigation, particularly electrodiagnostic testing, is mandatory.

The EMG pattern observed here (diffuse denervation in limb muscles with intact paraspinals) is the cornerstone for localizing the pathology to the anterior horn cell [11,12]. This pattern should prompt expedited genetic testing for SMN1 deletions, bypassing unnecessary additional imaging or invasive procedures.

Mild CK elevation is another potential red herring. While often thought to indicate myopathy, chronic denervation can also raise CK, typically in the mild‑to‑moderate range [8-10]. The combination of elevated CK with a normal aldolase provides an early clue pointing away from primary muscle disease. In our patient, this dissociation was present but initially overlooked.

The positive family history was the single most powerful shortcut to the diagnosis. In autosomal recessive disorders, an affected sibling is a major clue. Its omission from the initial workup represents a missed opportunity. Clinicians evaluating any patient with unexplained proximal weakness should routinely inquire about a family history of similar symptoms or neuromuscular disease.

The therapeutic landscape for SMA has changed dramatically. Three agents, nusinersen, risdiplam, and onasemnogene abeparvovec, are now available. Pivotal trials have demonstrated that nusinersen improves motor function in later‑onset SMA [15], and real‑world adult cohorts confirm stabilization or improvement with these therapies [14]. Therefore, a delayed diagnosis is no longer merely an academic problem; it directly affects access to therapies that may preserve ambulation and quality of life.

Limitations

As with any single case report, these findings should be interpreted within the context of the broader literature, and the diagnostic approach described should be validated in larger prospective studies. Electrodiagnostic tracings were not available for publication, which limits the visual documentation of the characteristic neurogenic changes described. A limitation of this report is the absence of clinical photographs of the lower extremities, as the patient declined to provide consent for their publication despite agreeing to the writing of the case report.

## Conclusions

SMA-4 should be considered in any adult presenting with slowly progressive, painless proximal leg weakness, even when the lumbar spine MRI shows degenerative stenosis that appears convincing. Incidental imaging abnormalities are common and must not override clinical reasoning. A structured diagnostic approach is essential: first, exclude common mimics with basic laboratory tests; second, perform EMG/NCS to localize the lesion; third, if the pattern suggests anterior horn cell disease (diffuse limb denervation with normal paraspinals), proceed directly to SMN1 genetic testing; and fourth, always inquire about family history.

In the era of disease‑modifying treatments, making the correct diagnosis promptly is essential to offering patients the best chance of preserving function. When clinical and imaging findings are discordant, clinicians must pursue further electrodiagnostic and genetic evaluation rather than attributing the presentation solely to incidental structural changes.
